# Predictors of vaccine hesitancy during the COVID-19 pandemic in Austria: A population-based cross-sectional study

**DOI:** 10.1007/s00508-022-02061-8

**Published:** 2022-08-10

**Authors:** Benedikt Till, Thomas Niederkrotenthaler

**Affiliations:** 1 Unit Suicide Research & Mental Health Promotion, Department of Social and Preventive Medicine, Center for Public Health, Medical University of Vienna, Kinderspitalgasse 15, 1090 Vienna, Austria; 2 Wiener Werkstaette for Suicide Research, Vienna, Austria

**Keywords:** COVID-19, Vaccination, Fear, Social media, Public health, Survey, Quota sampling

## Abstract

**Background:**

Unwillingness to get vaccinated against the coronavirus disease 2019 (COVID-19) is a major barrier in managing the pandemic. Previous studies have explored predictors of hesitancy to be vaccinated against COVID-19, but evidence on these predictors was partly mixed, and the number of assessed predictors was often limited. This study aimed to explore a wide range of potential predictors of vaccine hesitancy in a population-based cross-sectional study.

**Methods:**

We assessed associations of vaccine hesitancy with individuals’ fears about the future, social media use, and sociodemographics in a hierarchical multiple regression analysis. Data were collected via online questionnaires in a population-based cross-sectional study with 4018 respondents representative of the Austrian adult population between October and December 2020.

**Results:**

Vaccine hesitancy was predicted by freedom-related fears (i.e., fears regarding the political situation, particularly loss of personal freedoms), but were negatively associated with health-related fears (i.e., fears about physical or mental health) and society-related fears (i.e., fears regarding societal issues such as solidarity, distance learning, and isolation). Social media use as well as female gender, younger age, lower education, lower income, and living in rural regions were further predictors of vaccine hesitancy.

**Conclusion:**

The study confirms that public health efforts targeting unvaccinated persons need to address freedom-related fears and social media discourse in order to improve vaccine uptake in the population. Particularly individuals in socially and economically disadvantaged groups and social media users need to be targeted to reduce vaccine hesitancy.

**Supplementary Information:**

The online version of this article (10.1007/s00508-022-02061-8) contains supplementary material, which is available to authorized users.

## Introduction

The onset of the coronavirus disease 2019 (COVID-19) pandemic along with the implementation of lockdowns, social and physical distancing, and stay at home orders has drastically altered peoples’ lives and impacted global, public, and private economy [1]. Since widespread vaccination is now an important strategy of managing COVID-19 transmissions [2], reducing vaccine hesitancy in the general population is essential for overcoming the pandemic.

Previous studies have predominantly explored sociodemographics as predictors of vaccine hesitancy and found that younger age, lower education, lower socioeconomic status, and living in rural regions were associated with hesitancy to vaccinate against COVID-19 [2–7]. So far, however, the majority of these studies have been conducted outside of Europe. Furthermore, there is also conflicting evidence about some identified predictors. For example, in studies conducted in Australia, the USA, Austria, and Oman, male gender was associated with intentions to get vaccinated against COVID-19 [4–6, 8], whereas in a sample of 1658 respondents from the general population in Bangladesh, men had more vaccine hesitancy than women [9].

Two recent studies from Austria have assessed associations of vaccine hesitancy with political beliefs and psychosocial concepts and identified distrust in authorities as important predictor for distrust in vaccines [6, 10]. There is, however, a paucity of research with regards to other potentially relevant factors. For example, even before the onset of the COVID-19 pandemic, health-related misinformation was widespread on social media [11], with more content available related to anti-vaccination than pro-vaccination [12]. This dangerous trend on social media seems to have continued if not increased during the COVID-19 pandemic [13, 14]. Accordingly, distrust in vaccination against COVID-19 may be higher in individuals who prefer social media as their source of information. Other factors such as pre-existing health problems [15] or specific fears, such as fear of infection with COVID-19 [16, 17] or fear of vaccination side effects [10], may also be important determinants of willingness to get vaccinated against COVID-19. Studies exploring associations of vaccine hesitancy with specific fears or preferences for social media are currently lacking. This study aimed to assess associations of hesitancy to get vaccinated against COVID-19 with specific fears related to COVID-19, preferences for social media, and sociodemographics in a large population-based sample.

## Methods

### Participants

We conducted a cross-sectional online study and collected data from participants in four consecutive waves (wave #1: October 9–21; wave #2: October 30–November 11; wave #3: November 20–28; wave #4: December 11–22) during the COVID-19 pandemic in 2020. The data analyzed in the current work were collected as part of a larger project aiming to explore and monitor mental health in Austria during the first 9 months of the pandemic with a total of 12 waves of data collection [18]. For each wave, we used quota sampling techniques to recruit a sample of approx. 1000 individuals representative of the Austrian population for individuals of 16 years and older in terms of gender, age, region of residence, and education. The final four waves of the survey included data on vaccine hesitancy, which were used in the present study.

Participants were recruited via email from an online panel of a professional marketing company consisting of 30,000 registered users. Each participant could only participate once within one wave but was able to participate in subsequent waves. A total of 27,786 panel members were invited to participate in the study, and 6049 individuals accepted the invitation and started the survey. Of these, 4018 participants (66.4%) completed the entire survey and were included in the statistical analysis. Informed consent was obtained from all participants, and the study was approved by the Research Ethics Board of the Medical University of Vienna (study protocol 1391/2020, 23 April 2020).

### Measures

#### Vaccine hesitancy

We assessed participants’ hesitancy to get vaccinated against COVID-19 with the following question: “If there is a vaccine for COVID-19 approved by authorities in Austria, would you get yourself vaccinated?” Please note that no vaccine was officially approved by authorities in Austria at the time of the survey. Respondents answered this item on a scale from 1 (I definitely would) to 7 (I would definitely not).

#### Biggest fears about the future

We asked participants to elaborate on their currently biggest fears about the future with an open-ended question.

#### Preferences for social media

We asked participants to complete a rank order question by ranking “social media”, “radio”, “television”, “printed newspaper”, and “online newspaper” from 1 (most often used media type) to 5 (least often used media type) to indicate which types of media they use most often. In a second step, we coded whether participants ranked “social media” as their most often used media type (yes = 1, no = 0).

#### Socio-demographics

We asked participants to indicate their gender (male, female, other genders), age (16–29, 30–39, 40–49, 50–59, 60–69, 70+ years), highest completed education level (below high school, high school, college/university), zip code (with an open-ended question), net household income per month (< 500 €, 501–1000 €, 1001–1500 €, 1501–2000 €, 2001–3000 €, 3001–4000 €, 4001–5000 €, > 5000 €), number of individuals currently living in their household (ranging from 1 to > 8), and if they have pre-existing mental health problems (yes/no) or a pre-existing somatic morbidity (yes/no).

### Coding

In order to assess if participants lived in an urban or rural region, we categorized participants with zip codes of cities with a population of 10,000 or above as participants living in urban regions (*1*) and all others as participants living in rural areas (*0*). Based on this categorization, 1781 participants (44.3%) were currently living in an urban region, 1621 participants (40.3%) were currently living in a rural area, and 616 participants (15.3%) did not provide a valid zip code. Household per capita income was calculated by dividing the net household income by the square root of household members [19]. We used this formula for “equalized household income” as recommended by the Organisation for Economic Co-operation and Development (OECD) in order to account for the fact that households vary greatly in size and that income needed to support a family does not increase linearly with the number of individuals living in a household [19].

Two independent coders read and categorized participants’ entries regarding their biggest fears about the future. A total of 3880 participants (96.6%) provided valid entries. Following basic principles of thematic analysis [20], we identified four continuously recurring fears related to COVID-19 in participants’ entries: Health-related fears (i.e., fears regarding physical health, mental health, and/or the health system; *n* = 1030, 25.6%), economy-related fears (i.e., fears regarding economy, finances, housing, and/or employment; *n* = 1570, 39.1%), freedom-related fears (i.e., fears regarding the political situation, particularly loss of personal freedoms; *n* = 603; 15.0%), and society-related fears (i.e., fears regarding societal issues such as solidarity, distance learning, and/or isolation; *n* = 481, 12.0%). A total of 782 participants (19.5%) did not indicate any fears related to COVID-19. Detailed code definitions and coding examples are provided in Supplementary Table 1. Intercoder reliability was assessed based on Krippendorff’s alpha of 250 entries (approx. 5%) that were analyzed by both coders and was > 0.80 for all 4 codes, which is commonly considered a high level of agreement [21].

### Data analysis

Hierarchical multiple regression analysis was used to predict vaccine hesitancy. We controlled for wave number (i.e., time) by including a dummy variable for each wave. These dummy variables were put in the first step of the model. Preference for social media and sociodemographics were included in the second step, and all four COVID-19-related fears (*yes* = 1, *no* = 0) were included in the third step. The analysis was weighted in terms of combinations across gender, age, region of residence (i.e., federal state in Austria), and education with the random iterative method (RIM).

## Results

Supplementary Table 2 provides an overview of the weighted and unweighted frequencies of all predictors across all 4018 participants. An overview of the results of the hierarchical multiple regression analysis exploring predictors of vaccine hesitancy is shown in Table 1. Vaccine hesitancy (*M* = 4.24, *SD* = 2.36) was predicted by female gender, young age, lower education, living in rural regions, lower income, preferences for social media as the main source of information, and freedom-related fears, and they were negatively associated with health-related and society-related fears.

**Table 1:** Results of hierarchical multiple linear regression analysis to predict vaccine hesitancy

Predictor	Step 1 (*R*^2^ = 0.00, *F* = 1.46^a^ )				Step 2 (*R*^2^ = 0.10, *F* = 29.75^***b^ , ∆*R*^2^ = 0.10, ∆ *F* = 38.19^***c^)				Step 3 (*R*^2^ = 0.14, *F* = 31.76^***d^, ∆*R*^2^ = 0.04, ∆ *F* = 34.47^***e^)			
	*B*	SE *B*	β	*t*	*B*	SE *B*	β	*t*	*B*	SE *B*	β	*t*
Wave #2 (Oct 30–Nov 11)	−0.16	0.11	−0.03	−1.40	−0.16	0.11	−0.03	1.50	−0.15	0.11	−0.03	−1.41
Wave #3 (Nov 20–Nov 28)	−0.23	0.11	−0.04	−2.05^*^	−0.28	0.11	−0.05	−2.56^*^	−0.23	0.11	−0.04	−2.19^*^
Wave #4 (Dec 11–Dec 22)	−0.14	0.11	−0.03	−1.19	−0.16	0.11	−0.03	−1.44	−0.09	0.11	−0.02	−0.81
Gender: Female	–	–	–	**–**	0.83	0.08	0.18	10.62^***^	0.85	0.08	0.18	11.05^***^
Gender: Other gender	–	–	–	–	0.97	0.99	0.02	0.97	1.01	0.98	0.02	1.04
Age	–	–	–	**–**	−0.21	0.03	−0.16	−7.99^***^	−0.17	0.03	−0.13	−6.54^***^
Education: High school	–	–	–	**–**	−0.29	0.11	−0.04	−2.60^**^	−0.31	0.11	−0.05	−2.80^**^
Education: University/college	–	–	–	**–**	−0.36	0.12	−0.05	−2.95^**^	−0.36	0.12	−0.05	−3.09^**^
Living in urban region	–	–	–	**–**	−0.23	0.08	−0.05	−2.95^**^	−0.22	0.08	−0.05	−2.86^**^
Household per capita income	–	–	–	–	−0.05	0.03	−0.03	−1.58	−0.08	0.03	−0.04	−2.33^*^
Pre-existing mental health problems	–	–	–	–	−0.21	0.12	−0.03	−1.80	−0.16	0.12	−0.02	−1.40
Pre-existing somatic morbidity	–	–	–	**–**	−0.14	0.09	−0.03	−1.59	−0.10	0.09	−0.02	−1.20
Social media as preferred source	–	–	–	**–**	0.31	0.09	0.06	3.36^***^	0.29	0.09	0.06	3.14^**^
Health-related fears	–	–	–	**–**	–	–	–	–	−0.68	0.10	−0.13	−7.09^***^
Economy-related fears	–	–	–	–	–	–	–	–	0.16	0.09	0.03	1.84
Freedom-related fears	–	–	–	**–**	–	–	–	–	0.72	0.11	0.11	6.62^***^
Society-related fears	–	–	–	**–**	–	–	–	–	−0.35	0.12	−0.05	−2.89^**^

Values are unstandardized (*B*) and standardized (β) regression coefficients, standard errors of the unstandardized regression coefficients (SE *B*), and t values (*t*) based on weighted data. Also reported are adjusted *R*^2^ and *F* values for each step of the model along with changes in *R*^2^ and *F* values (∆*R*^2^, ∆*F*) from step 1 to step 2 and step 2 to step 3; ^*^p < 0.05; ^**^p < 0.01; ^***^p < 0.001 (two-tailed). Significant *p*-values (< 0.05) are in bold.

^a^*df*_1_ = 3, *df*_2_ = 3406

^b^*df*_1_ = 13, *df*_2_ = 3396

^c^*df*_1_ = 10, *df*_2_ = 3396

^d^*df*_1_ = 17, *df*_2_ = 3392

^e^*df*_1_ = 4, *df*_2_ = 3392

We conducted a sensitivity analysis to check whether the findings were different if unweighted instead of weighted data were used in the statistical analyses. Results of the regression analysis with unweighted data, which can be found in Supplementary Table 3, were similar to those with weighted data.

## Discussion

The findings of the current study showed that hesitancy to get vaccinated against COVID-19 was associated with specific COVID-19-related fears. Whereas having health-related or society-related fears was associated with higher willingness to get vaccinated against COVID-19, freedom-related fears predicted vaccine hesitancy. Individuals with fears about losing personal freedoms obviously were less worried about COVID-19 per se, but more about the consequences of measures such as lockdowns, governmental restrictions, and stay-at-home orders on autonomy and personal rights and liberties, including laws on mandatory vaccination. These findings highlight that public health efforts targeting unvaccinated individuals need to address fears of loss of personal freedoms in promotion campaigns. Although some suggestions have been made accordingly, current governmental responses to vaccine hesitancy have not comprehensively addressed these fears of loss of personal freedoms. In several European countries, including Austria, official information on vaccination efficacy and side effects were communicated unclearly and consisted mainly of fact checking [22], and public discussions focused primarily on whether a general vaccination mandate can be ethically justified [23]. More governmental efforts to listen and cater to unvaccinated people’s concerns about vaccines against COVID-19 as recommended by the OECD [24] are needed.

We also found that preference for social media predicted vaccine hesitancy. This is consistent with previous studies that observed an emergence of fake news on COVID-19, in particular with regards to vaccination, on social media [13, 14]. This finding highlights that the reliance on social media as the main source of information may play a role in developing or strengthening hesitancy to get vaccinated against COVID-19, although a definite statement on causation cannot be made based on the cross-sectional nature of our study.

Consistent with previous findings [2–8], female gender, younger age, lower education and socioeconomic status, and living in rural regions predicted vaccine hesitancy. Importantly, these particular groups have also been identified as those who have been affected the most by the pandemic with regards to their mental health in Austria [18]. Individuals in these groups had higher scores in terms of suicidal thoughts, depressive symptoms, anxiety, and domestic violence [18]. Public health campaigns aiming to improve willingness to get vaccinated against COVID-19 need to find ways to tailor their messages to these specific groups.

### Strengths and limitations

The collection of data from a large quota-based sample that was representative of the Austrian adult population in terms of gender, age, education, and region of residence, was a strength of the current study. This was also the first study that assessed specific fears related to COVID-19 with a content analysis.

There are also some limitations. First, the cross-sectional design prevented us from inferring causality for the identified associations. Furthermore, the measure assessing vaccine hesitancy was a single item, and no vaccine had been officially approved by authorities at the time of the survey. Also, some respondents participated in more than one wave of data collection in this study; however, in sensitivity analyses, discrepancies in patterns were small when only participants were included who indicated that they did not participate in previous waves of the current study (data available upon request) [18].

## Conclusion

The findings of the current study show that COVID-19-related fears with regards to loss of personal freedoms are associated with hesitancy to get vaccinated against COVID-19. Vaccine promotion campaigns targeting unvaccinated persons by governmental and public health agencies should address fears about restrictions of personal freedoms to establish trust and reduce vaccine hesitancy. These efforts should be delivered particularly via social media and need to be tailored to young people, women, rural residents, and socially or economically disadvantaged groups (e.g., individuals with low income or low education) who have been shown to suffer most in terms of mental ill health during the pandemic.

## Supplementary Information

Supplementary Tables 1–3

## References

[1] Xiong J, Lipsitz O, Nasri F, Lui LMW, Gill H, Phan L (2020). Impact of COVID-19 pandemic on mental health in the general population: a systematic review. J Affect Disord.

[2] Paul E, Steptoe A, Fancourt D (2021). Attitudes toward vaccines and intervention to vaccinate against COVID-19: Implications for public health communications. Lancet Reg Health Eur.

[3] Danabal KGM, Magesh SS, Saravanan S, Gopichandran V (2021). Attitude towards COVID 19 vaccines and vaccine hesitancy in urban and rural communities in Tamil Nadu, India—a community based survey. BMC Health Serv Res.

[4] Enticott J, Gill JS, Bacon SL, Lavoie KL, Epstein DS, Dawadi S (2022). Attitudes towards vaccines and intention to vaccinate against COVID-19: a cross-sectional analysis—implications for public health communications in Australia. BMJ Open.

[5] Fisher KA, Bloomstone SJ, Walder J, Crawford S, Fouayzi H, Mazor KM (2020). Attitudes toward a potential SARS-CoV-2 vaccine: a survey of U.S. adults. Ann Intern Med.

[6] Schernhammer E, Weitzer J, Laubichler MD, Birmann BM, Bertau M, Zenk L (2022). Correlates of COVID-19 vaccine hesitancy in Austria: trust and the government. J Public Health.

[7] Shaw J, Stewart T, Anderson KB, Hanley S, Thomas SJ, Salmon DA, Morley C (2021). Assessment of US healthcare personnel attitudes towards Coronavirus disease 2019 (COVID-19) vaccination in a large university healthcare system. Clin Infect Dis.

[8] Khamis F, Badahdah A, Mahyijari NA, Lawati FA, Noamani JA, Salmi IA, Bahrani MA (2022). Attitudes towards COVID-19 vaccine: a survey of health care workers in Oman. J Epidemiol Glob Health.

[9] Islam MS, Siddique AB, Akter R, Tasnim R, Sujan MSH, Ward PR, Sikder MT (2021). Knowledge, attitudes and perceptions towards COVID-19 vaccinations: a cross-sectional community survey in Bangladesh. BMC Public Health.

[10] Stamm TA, Partheymüller J, Mosor E, Ritschl V, Kritzinger S, Eberl J-M (2022). Coronavirus vaccine hesitancy among unvaccinated Austrians: assessing underlying motivations and the effectiveness of interventions based on a cross-sectional survey with two embedded conjoint experiments. Lancet Reg Health Eur.

[11] Stecula DA, Kuru O, Jamieson KH (2020). How trust in experts and media use affect acceptance of common anti-vaccination claims. Harv Kennedy Sch (HKS) Misinf Rev.

[12] Lahouati M, De Coucy A, Sarlangue J, Cazanave C (2020). Spread of vaccine hesitancy in France: What about YouTube?. Vaccine.

[13] Carrion-Alvarez D, Tijerina-Salina PX (2020). Fake news in COVID-19: a perspective. Health Promot Perspect.

[14] Schuetz SW, Sykes TA, Venkatesh V (2021). Combating COVID-19 fake news on social media through fact checking: antecedents and consequences. Eur J Inf Syst.

[15] Batty DG, Deary IJ, Altschul D (2022). Pre-pandemic mental and physical health as predictors of COVID-19 vaccine hesitancy: evidence from a UK-wide cohort study. Ann Med.

[16] Hakansson A, Claesdotter E (2022). Fear of COVID-19, compliance with recommendations against virus transmission, and attitudes towards vaccination in Sweden. Heliyon.

[17] Sekizawa Y, Hashimoto S, Denda K, Ochi S, So M (2022). Association between COVID-19 vaccine hesitancy and generalized trust, depression, generalized anxiety, and fear of COVID-19. BMC Public Health.

[18] Niederkrotenthaler T, Laido Z, Kirchner S, Braun M, Metzler H, Waldhör T (2022). Mental health over nine months during the SARS-CoV2 pandemic: representative cross-sectional survey in twelve waves between April and December 2020 in Austria. J Affect Disord.

[19] Keeley B (2015). Income inequality: the gap between rich and poor.

[20] Braun V, Clarke V (2006). Using thematic analysis in psychology. Qual Res Psychol.

[21] Krippendorff K (2004). Content analysis: an introduction to its methodology.

[22] Heiss R, Waser M, Falkenbach M, Eberl J‑M. How have governments and public health agencies responded to misinformation during the COVID-19 pandemic in Europe? European Observatory on Health Systems and Policies. 2021. https://eurohealthobservatory.who.int/monitors/hsrm/analyses/hsrm/how-have-governments-and-public-health-agencies-responded-to-misinformation-during-the-covid-19-pandemic-in-europe. Accessed 8 June 2022.

[23] Druml C, Czech H (2022). A pandemic is no private matter: the COVID-19 vaccine mandate in Austria. Lancet Respir Med.

[24] Organisation for Economic Co-operation and Development (2021). Enhancing public trust in COVID-19 vaccination: the role of governments.

