# Supplementary material for: Predictors of vaccine hesitancy during the COVID-19 pandemic in Austria: A population-based cross-sectional study
Source: Wien Klin Wochenschr. 2022 Aug 10;134(23-24):822–7. doi: 10.1007/s00508-022-02061-8 (PMC9364912; doi:10.1007/s00508-022-02061-8)
Supplement: Supplementary file 1 — Supplementary Tables 1–3 [file 508_2022_2061_MOESM1_ESM.docx]

Supplementary Table 1. *Coding system for content analysis of COVID-19-related fears*

| **Code** | **Definition** | **Coding examples** |
| --- | --- | --- |
| *Health* | References of concerns about the future with regard to individuals’ physical and mental health, including concerns about the progression of the COVID-19 pandemic, its containment (e.g. via vaccination), and its negative consequences for the healthcare system. | “Due to my severe pre-existing conditions, I am a high-risk patient and therefore take extra care to avoid an infection”  “...that there will be no vaccine in the near future and the current situation will not improve” |
| *Economy* | References of concerns about the future with regard to the economy, finances, housing, and employment (on either individual, societal, or global level). | “… that it hits the economy so badly that I lose my job and there will be even more unemployed people”  “...that my husband and I can no longer pay the mortgage for our house” |
| *Freedom* | References of concerns about the future with regard to the political situation and administrative management, particularly about limitations of fundamental rights and freedoms due to restrictive policies in order to contain the COVID-19 pandemic. | “The transformation of democracy into an authoritarian state”  “... that events/trips/etc. won’t be possible for a long time”  “Governmental actions often target the wrong areas” |
| *Society* | References of concerns about the future with regard to any societal issues. This includes concerns related to social coexistence during the COVID-19 pandemic (e.g. lack of solidarity and responsibility among fellow citizens), but also topics such as social distancing and its impact on society (e.g., social isolation, issues with passing an exam or finishing school). | “Impact of social distancing on children/youth and their future”  “Increasing carelessness of my fellow citizens”  “How we can get through the final exam smoothly with all the weeks in distance learning” |

Supplementary Table 2. *Overview of all predictors across all participants (n=4,018)*

| **Characteristic** | **Weighted, *n* (%)** | **Unweighted, *n* (%)** |
| --- | --- | --- |
| *Wave* |  |  |
| Wave #1 (Oct 09 – Oct 21) | 1,007 (25.1) | 1,007 (25.1) |
| Wave #2 (Oct 30 – Nov 11) | 1,005 (25.0) | 1,005 (25.0) |
| Wave #3 (Nov 20 – Nov 28) | 1,004 (25.0) | 1,004 (25.0) |
| Wave #4 (Dec 11 – Dec 22) | 1,002 (24.9) | 1,002 (24.9) |
| *Gender* |  |  |
| Male | 1,942 (48.3) | 1,939 (48.3) |
| Female | 2,068 (51.6) | 2,071 (51.5) |
| Other genders | 8 (0.2) | 8 (0.2) |
| *Age group* |  |  |
| 16-29 | 832 (20.7) | 799 (19.9) |
| 30-39 | 629 (15.7) | 645 (16.1) |
| 40-49 | 787 (19.6) | 627 (15.6) |
| 50-59 | 651 (16.2) | 740 (18.4) |
| 60-69 | 501 (12.5) | 536 (13.3) |
| 70+ | 618 (15.4) | 671 (16.7) |
| *Highest completed education level* |  |  |
| Below high school | 2,993 (74.5) | 2,996 (74.6) |
| High school | 551 (13.7) | 556 (13.8) |
| College/university | 474 (11.8) | 466 (11.6) |
| Living in urban region | 1,781 (44.3) | 1,808 (45.0) |
| *Monthly net income (in Euro)* |  |  |
| <500 | 199 (5.0) | 191 (4.8) |
| 501-1000 | 445 (11.1) | 440 (11.0) |
| 1001-1500 | 573 (14.3) | 576 (14.3) |
| 1501-2000 | 718 (17.9) | 714 (17.8) |
| 2001-3000 | 939 (23.4) | 945 (23.5) |
| 3001-4000 | 664 (16.5) | 674 (16.8) |
| 4001-5000 | 290 (7.2) | 288 (7.2) |
| >5000 | 189 (4.7) | 190 (4.7) |
| *Number of household members* |  |  |
| 1 | 997 (24.8) | 1,005 (25.0) |
| 2 | 1,536 (38.2) | 1,579 (39.3) |
| 3 | 686 (17.1) | 673 (16.7) |
| 4 | 538 (13.4) | 514 (12.8) |
| 5 | 173 (4.3) | 164 (4.1) |
| 6 | 53 (1.3) | 50 (1.2) |
| 7 | 20 (0.5) | 19 (0.4) |
| 8 | 12 (0.3) | 11 (0.3) |
| >8 | 3 (0.1) | 3 (0.1) |
| Pre-existing mental health problems | 572 (14.2) | 566 (14.1) |
| Pre-existing somatic morbidity | 1,205 (30.0) | 1,228 (30.6) |
| *COVID-19-related fears* |  |  |
| Health | 1,030 (25.6) | 1,049 (26.1) |
| Economy | 1,570 (39.1) | 1,556 (38.7) |
| Freedom | 603 (15.0) | 597 (14.9) |
| Society | 481 (12.0) | 480 (11.9) |

Values are weighed und unweighted absolute (*n*) and relative (%) frequencies of all predictors.

Supplementary Table 3. *Results of hierarchical multiple linear regression analysis to predict vaccine hesitancy*

| **Predictor** | **Step 1**  (*R*^2^=.00, *F*=1.67^a^) | | | | **Step 2**  (*R*^2^=.10, *F*=**30.23^***b^**, Δ*R*^2^=.10, Δ*F*=**38.74^***c^**) | | | | **Step 3**  (*R*^2^=.14, *F*=**32.14^***d^**, Δ*R*^2^=.04, Δ*F*=**34.49^***e^**) | | | |
| --- | --- | --- | --- | --- | --- | --- | --- | --- | --- | --- | --- | --- |
|  | *B* | SE *B* | β | *t* | *B* | SE *B* | β | *t* | *B* | SE *B* | β | *t* |
| Wave #2 (Oct 30 – Nov 11) | -.17 | .11 | -.03 | -1.46 | -.17 | .11 | -.03 | 1.59 | -.16 | .11 | -.03 | -1.47 |
| Wave #3 (Nov 20 – Nov 28) | -.25 | .11 | -.05 | **-2.20^*^** | -.30 | .11 | -.06 | **-2.71^**^** | -.25 | .11 | -.05 | **-2.33^*^** |
| Wave #4 (Dec 11 – Dec 22) | -.15 | .11 | -.03 | -1.30 | -.17 | .11 | -.03 | -1.59 | -.10 | .11 | -.02 | -0.93 |
| Gender: Female |  |  |  |  | .81 | .08 | .17 | **10.38^***^** | .84 | .08 | .18 | **10.87^***^** |
| Gender: Other gender |  |  |  |  | .98 | 1.00 | .02 | 0.99 | 1.03 | .98 | .02 | 1.05 |
| Age |  |  |  |  | -.21 | .03 | -.16 | **-8.10^***^** | -.17 | .03 | -.13 | **-6.66^***^** |
| Education: High school |  |  |  |  | -.29 | .11 | -.04 | **-2.62^**^** | -.31 | .11 | -.05 | **-2.82^**^** |
| Education: University/college |  |  |  |  | -.35 | .12 | -.05 | **-2.89^**^** | -.36 | .12 | -.05 | **-3.00^**^** |
| Living in urban region |  |  |  |  | -.24 | .08 | -.05 | **-3.07^**^** | -.23 | .08 | -.05 | **-3.01^**^** |
| Household per capita income |  |  |  |  | -.06 | .03 | -.03 | -1.84 | -.09 | .03 | -.04 | **-2.56^*^** |
| Pre-existing mental health problems |  |  |  |  | -.23 | .12 | -.03 | **-2.00^*^** | -.19 | .12 | -.03 | -1.63 |
| Pre-existing somatic morbidity |  |  |  |  | -.15 | .09 | -.03 | -1.76 | -.11 | .09 | -.02 | -1.32 |
| Social media as preferred source |  |  |  |  | .31 | .09 | .06 | **3.27^**^** | .27 | .09 | .05 | **2.99^**^** |
| Health-related fears |  |  |  |  |  |  |  |  | -.69 | .10 | -.13 | **-7.26^***^** |
| Economy-related fears |  |  |  |  |  |  |  |  | .13 | .09 | .03 | 1.53 |
| Freedom-related fears |  |  |  |  |  |  |  |  | .72 | .11 | .11 | **6.53^***^** |
| Society-related fears |  |  |  |  |  |  |  |  | -.35 | .12 | -.05 | **-2.90^**^** |

Values are unstandardized (*B*) and standardized (β) regression coefficients, standard errors of the unstandardized regression coefficients (SE *B*), and t values (*t*) based on unweighted data. Also reported are adjusted *R*^2^- and *F*-values for each step of the model along with changes in *R*^2^- and *F*-values (Δ*R*^2^, Δ*F*) from step 1 to step 2 and step 2 to step 3; ^*^*p* < .05; ^**^*p* < .01; ^***^*p* < .001 (two-tailed). Significant p-values (< .05) are in bold.

^a^*df*_1_ = 3, *df*_2_ = 3406; ^b^*df*_1_ = 13, *df*_2_ = 3396; ^c^*df*_1_ = 10, *df*_2_ = 3396; ^d^*df*_1_ = 17, *df*_2_ = 3392; ^e^*df*_1_ = 4, *df*_2_ = 3392.
